# Mitigating the threat of “Disease X” to global health security

**DOI:** 10.1016/j.nmni.2024.101223

**Published:** 2024-01-26

**Authors:** Emery Manirambona, Olalekan John Okesanya, Deborah Oluwaseun Shomuyiwa, Shuaibu Saidu Musa, Don Eliseo Lucero-Prisno III

**Affiliations:** Global Health Focus, Bujumbura, Burundi; Faculty of Laboratory Hygiene and Epidemiology, University of Thessaly, Greece; Faculty of Pharmacy, University of Lagos, Lagos, Nigeria; Department of Nursing Science, Ahmadu Bello University, Zaria, Nigeria; Department of Global Health and Development, London School of Hygiene and Tropical Medicine, London, United Kingdom

**Keywords:** Disease X, Emerging infections, Outbreaks preparedness, Readiness

Dear Editor,

Global public health security seeks to create a safer future for all people through coordinated global public health initiatives. However, the evolution of different zoonotic viruses has revealed the profound threat to global public health security, emphasising the possibility of massive dreadful outbreaks [1]. The evolution of the virosphere has been accounted for using comparative genomics and metaviromics advances, predicting the global virome to be between 10^7^ and 10^9^ different virus species or more billions [2]. Many of these species are also projected to be pathogenic to humans, raising concerns about the constant chance of encountering new and possibly harmful infectious agents [1]. This growing awareness prompts a closer look at the concept of “Disease X'', an enigmatic term that describes the hypothetical emergence of a novel and highly infectious pathogen with pandemic potential [1]. “Disease X '' is a crucial component in the range of potential global health hazards. According to the World Health Organisation (WHO), it represents the recognition that a significant international epidemic could be caused by a virus that is yet to be recognised as the cause of human disease. This enigmatic category spans a wide range of pathogens, including viruses, bacteria, fungi, parasites, and prions, including drug-resistant bacteria strains, accounting for roughly 54 % of known emerging infectious disease occurrences between 1940 and 2004 [3].

The recent experience demonstrated that the effects of new infectious diseases have been disproportionately severe in recent tragic events attributed to RNA viruses including the introduction of influenza H1N1 and H5N1, SARS-CoV, Ebola virus, Lassa virus and MERS-CoV [4]. These infections have an important ability to replicate across multiple host species, facilitated by their reverse transcriptase, resulting in great mutability and evasion of host immune responses. Surprisingly, 94 % of zoonotic viruses impacting humans are RNA viruses, highlighting the importance of knowing and monitoring this specific group in ongoing efforts to avoid and reduce new infectious illnesses. According to Iserson K's research, equally important is the growing concern extending to the potential introduction of engineered germs, whether through laboratory accidents or bioterrorism, adding to the looming prospect of a deadly Disease X.

Disease X was added to the WHO's priority list in 2018 to promote dialogue and readiness for future global pandemics, as well as to address the evolving risk of Disease X, which has yet to be discovered [1]. World leaders later convened at the World Economic Forum to examine this potential threat and emphasised that it could be more fatal than the recent Covid-19 pandemic. This highlights the importance of devising intricate new protective measures to prevent new deadly local, regional and international epidemics. The effective preparedness should focus on the innovative measures needed to prepare healthcare systems for the new challenges that may be posed by a potentially more lethal pandemic.

Developing international guidelines for bioterrorism control and active surveillance must be strengthened. Global collaboration among scientists and clinicians must also be promoted for a comprehensive screening, testing and aggressive contact tracing. Recognising the rapid zoonotic spillover caused by the rising human global footprint and transportation networks, it is critical to match intrinsic host-pathogen interactions with extrinsic environmental and socioeconomic factors. Addressing host behaviour, susceptibility, and the effects of urbanisation, climate change and land use changes is critical to effectively reducing the risk of spillover and human disease development [1,5].

Furthermore, a comprehensive One Health approach is required to prepare for Disease X, which includes three critical tactics. First, optimise wildlife-livestock-human surveillance in growing infectious disease hotspots by focusing on targeted and efficient surveillance, increasing capacity, and collaborating internationally. Second, perform basic and translational research to improve vaccine and therapy development, with an emphasis on identifying novel viruses, understanding transmission factors, and generating broad-spectrum medicines [5]. Third, decrease spillover risk and spread by minimising risky behaviours, enforcing rules, and encouraging sustainable food production. This method acknowledges the difficulties in predicting viral zoonotic potential and emphasises multidisciplinary collaboration across disciplines such as ecology, public health, virology, and sociology. Furthermore, the problematic use of regulated gene drive techniques for vector/pathogen control should be addressed [1,5].

All nations must take a proactive strategy to detect and address the root causes and evolution of emerging infectious diseases (EIDs) earlier in the emergence chain in order to slow the virus's spread and reduce their effect on population, while lessening the strain on the healthcare system. Finally, successful measures used to tackle the COVID-19 pandemic can serve as a foundation to set new safeguarding initiatives, thus preserving global health security.

## Ethics approval and consent to participate

Not applicable.

## Consent for publication

Not applicable.

## Availability of data and material

Not applicable.

## Funding

None.

## CRediT authorship contribution statement

**Emery Manirambona:** Conceptualization, Data curation, Formal analysis, Methodology, Project administration, Supervision, Validation, Visualization, Writing – original draft, Writing – review & editing. **Olalekan John Okesanya:** Data curation, Methodology, Visualization, Writing – original draft, Writing – review & editing. **Deborah Oluwaseun Shomuyiwa:** Data curation, Validation, Writing – original draft, Writing – review & editing. **Shuaibu Saidu Musa:** Data curation, Validation, Writing – original draft, Writing – review & editing. **Don Eliseo Lucero-Prisno III:** Data curation, Validation, Writing – original draft, Writing – review & editing.

## Declaration of competing interest

The authors declare that they have no known competing financial interests or personal relationships that could have appeared to influence the work reported in this paper.
